# The role of artificial intelligence in drug screening, drug design, and clinical trials

**DOI:** 10.3389/fphar.2024.1459954

**Published:** 2024-11-29

**Authors:** Yuyuan Wu, Lijing Ma, Xinyi Li, Jingpeng Yang, Xinyu Rao, Yiru Hu, Jingyi Xi, Lin Tao, Jianjun Wang, Lailing Du, Gongxing Chen, Shuiping Liu

**Affiliations:** ^1^ School of Pharmacy, Hangzhou Normal University, Hangzhou, Zhejiang, China; ^2^ Key Laboratory of Elemene Class Anti-Cancer Chinese Medicines, Engineering Laboratory of Development and Application of Traditional Chinese Medicines, Collaborative Innovation Center of Traditional Chinese Medicines of Zhejiang Province, Hangzhou Normal University, Hangzhou, Zhejiang, China; ^3^ Department of Respiratory Medicine of Affiliated Hospital, Hangzhou Normal University, Hangzhou, Zhejiang, China; ^4^ Key Laboratory of Pollution Exposure and Health Intervention of Zhejiang Province, Shulan International Medical College, Zhejiang Shuren University, Hangzhou, China

**Keywords:** artificial intelligence, drug discovery, drug screening, drug design, clinical trials

## Abstract

The role of computational tools in drug discovery and development is becoming increasingly important due to the rapid development of computing power and advancements in computational chemistry and biology, improving research efficiency and reducing the costs and potential risks of preclinical and clinical trials. Machine learning, especially deep learning, a subfield of artificial intelligence (AI), has demonstrated significant advantages in drug discovery and development, including high-throughput and virtual screening, *ab initio* design of drug molecules, and solving difficult organic syntheses. This review summarizes AI technologies used in drug discovery and development, including their roles in drug screening, design, and solving the challenges of clinical trials. Finally, it discusses the challenges of drug discovery and development based on AI technologies, as well as potential future directions.

## 1 Introduction

New drug development includes the discovery of drug lead compounds and drug optimization, which is a long, expensive, and high-risk process. The transformation of a new drug from a promising candidate to a marketable product can take over a decade or more, cost up to a billion dollars, and result in a high rate of clinical failure (Liu C. et al., 2021). Currently, diseases such as cancer, diabetes, Alzheimer’s disease, and Parkinson’s disease significantly affect human health and have become a serious public health problem globally, making drug discovery and development increasingly critical (Yuan et al., 2023; Zheng and Jiang, 2019). Drug development companies have adopted various methods to overcome this dilemma, with artificial intelligence (AI) playing a key role. For example, a study by the technology company Tech Emergence revealed that applying AI to new drug development can speed up the process by 2%, and a report by Goldman Sachs predicted that as AI technology matures, the annual savings in the field of new drug development could be as high as 28 billion dollars (Mao and Liu, 2021). The number of innovative drugs approved in China’s drug market was 14, 24, 18, 37, 49, 48, and 47 between 2014 and 2020, with the number of domestically produced innovative drugs increasing from 0 in 2017 to 14 in 2020 (Tang et al., 2022). These figures are attributed to the undeniable influence of AI on this process, indicating that AI is anticipated to revolutionize drug development.

## 2 Technologies and algorithms related to AI in drug discovery and development

The concept of AI dates back to 1950, when scientist Alan Turing described a simple test, later known as the “Turing Test” in his book Computing Machinery and Intelligence, to determine whether a computer exhibited human intelligence. He described AI as similar to but more complex than the human brain. Turing is thus known as the “Father of AI” (Mintz and Brodie, 2019; Kaul et al., 2020).

Machine learning (ML) is a subfield of AI, with deep learning (DL) as a subset of ML (Pandiyan and Wang, 2022). Currently, AI can analyze more complex algorithms and perform DL. Many related algorithmic models have been developed for drug discovery. ML algorithms have been used in several drug discovery processes, including peptide synthesis, structure-based virtual screening, ligand-based virtual screening, toxicity prediction, drug monitoring and release, pharmacodynamic modeling, quantitative structure-activity relationships, drug repositioning, polypharmacology, and physicochemical activities (Gupta et al., 2021). The relevant algorithmic models based on ML are described below.

### 2.1 ML

**FIGURE 1:**
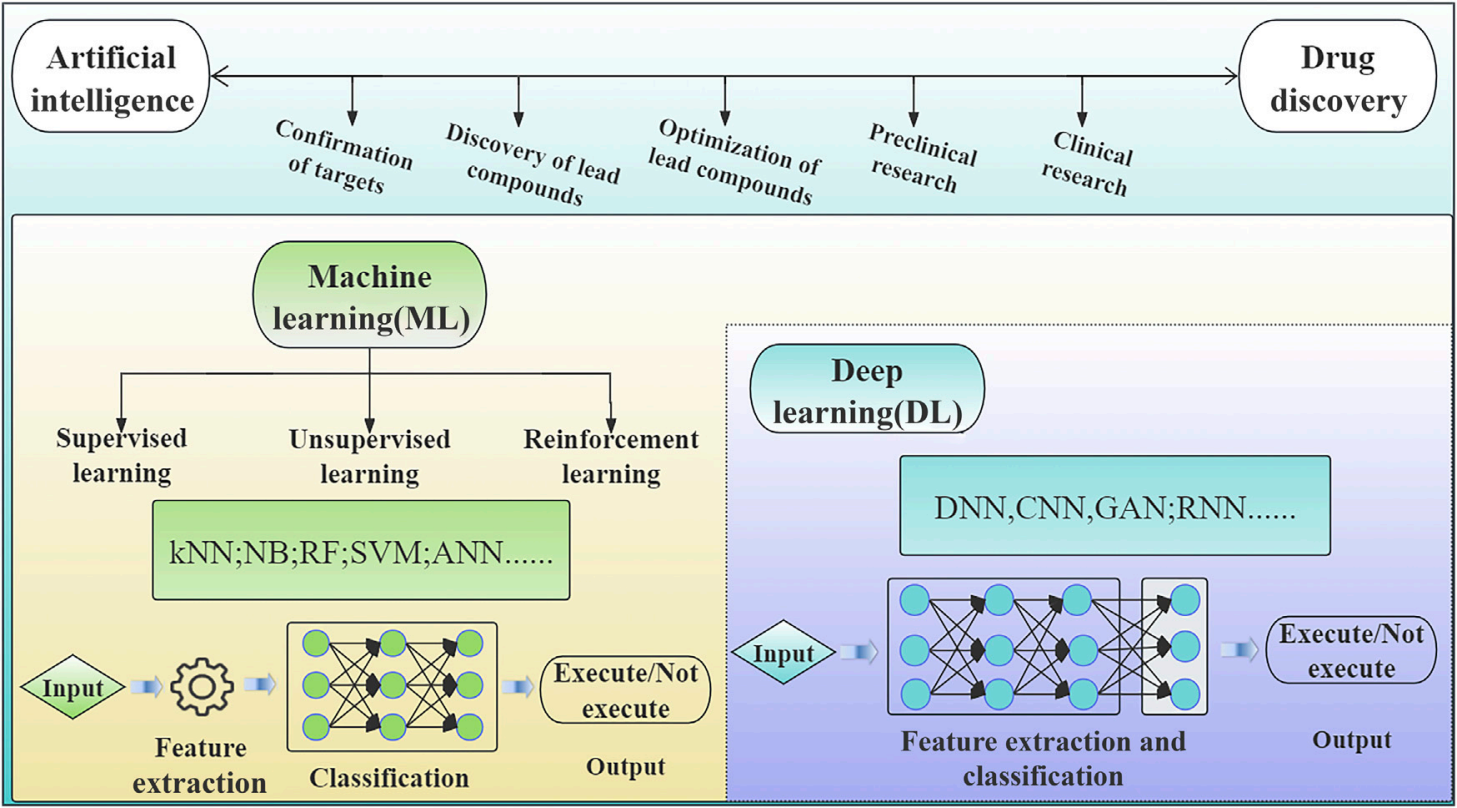
An overview of AI applications and technologies in drug discovery. The use of AI in drug discovery encompasses various applications and techniques The integration of AI into drug discovery involves the incorporation of ML and DL, with DL being a subset of ML. ML is further classified into three primary types: supervised, unsupervised, and reinforcement learning. This figure also delineates the ML and DL algorithms, highlighting the differences in their execution methodologies.

ML refers to AI algorithms in which models are trained on large datasets to learn rules, analyze new data, and make predictions and decisions. There are three main types of ML: supervised learning, unsupervised learning, and reinforcement learning (Figure 1) (Pandiyan and Wang, 2022; Tuan et al., 2023; Mak and Pichika, 2019). Supervised learning involves training algorithms on labeled datasets with predetermined correct answers for each input, enabling accurate predictions of new, unseen inputs (Islam et al., 2024). Unsupervised learning recognizes hidden patterns in data, clusters them, and interprets them in groups, with outputs such as disease subtypes and target discovery (Mak and Pichika, 2019). Reinforcement learning entails learning from interactions with the environment (Islam et al., 2024). For instance, Chen et al. used the Trinity software to assemble 148,784 transcripts and 78,092 single genes from clean reads. Expression patterns or functionally relevant gene clusters could be identified under specific conditions by analyzing the assembled gene expression data using the ML approach (Chen et al., 2021).

DL, a ML technique based on various types of neural networks (NNs) that utilize a hierarchical structure to learn more complex structures and relationships in a dataset (Smith et al., 2023), is a type of modeling and learning that employs neuronal structures mimicking biological neural networks (Figure 1). DL differs from ML because it utilizes data types and learning methods more adept at handling massive, high-dimensional, and complex data structures (Lac et al., 2024). DL eliminates some of the data preprocessing steps associated with ML and continuously corrects prediction errors through gradient descent and backpropagation. DL techniques have demonstrated significant potential and prospects for various clinical applications, including medical image analysis, disease diagnosis, treatment prediction, and patient monitoring (Wang et al., 2024).

### 2.2 Traditional ML algorithms

The most common ML algorithms in drug discovery and development include k-Nearest Neighbors (kNN), Naïve Bayesian Classifier (NB), Random Forest (RF), Support Vector Machine (SVM), and Artificial Neural Networks (ANNs) (Figure 1). Their roles in drug discovery and development have been summarized as following.

#### 2.2.1 kNN

kNN implies that a sample belongs to a specific category if the majority of the k-nearest samples (the closest neighbors in the feature space) surrounding it belong to that category (Guan et al., 2023). In recent studies, Yang M et al. used the weighted kNN (WkNN) method to improve the overall density of the drug-disease association matrix based on the kNN principle for drug repositioning research (Yang et al., 2023).

#### 2.2.2 NB

NB is one of the Bayesian classifiers that can be used to train a model with a dataset of known categories, enabling the categorization of data from unknown categories (Yang et al., 2023). NB has been used in the pharmaceutical field due to its simplicity, speed, and effectiveness. For instance, based on the principle of NB, Shi H et al. trained a classifier to recognize positive and negative samples of the pregnane X receptor (PXR). This classifier was then used to distinguish between PXR activators and non-activators, thereby improving classification efficiency (Shi et al., 2015).

#### 2.2.3 RF

The RF is a regression tree technique that uses bootstrap aggregation and randomization of predictor variables to achieve a high degree of predictive accuracy (Rigatti, 2017). Recently, Ryu et al. (2022) developed a computational model called PredMS based on the RF model to predict the stability of small compounds during metabolism.

#### 2.2.4 SVM

The SVM is a two-class classification model. Its basic model is defined as a linear classifier that maximizes the intervals on the feature space. The learning strategy of SVM is interval maximization, which ultimately translates to solving a convex quadratic programming problem (Yang et al., 2023). It is crucial for predicting molecular interactions, binding affinity, and other properties between ligands and target proteins (Shiammala et al., 2023). Jing-Fang Z et al. selected 324 neurotoxic compounds and 234 non-neurotoxic compounds based on the combination of SVM with Cfs subset evaluation and Best First-D1-N5 search using a web database. Compounds were used as the dataset for constructing the neurotoxicity discriminant model. This dataset included charge distribution and physicochemical and geometric descriptors to characterize the molecular structures of neurotoxic compounds. The accuracy, sensitivity, and specificity were >80% (Zhang et al., 2014).

#### 2.2.5 ANNs

ANNs are computer programs that simulate the operation of many processing units that mimic nerve cells and the basic biological mechanisms by which they connect and interact with each other. ANNs are a subset of ML that were created as direct analogs of biological NNs (Shiammala et al., 2023). Like the human brain, ANNs can learn from experiences and understand the general relationships between variables. These algorithms are crucial for various processes, including drug screening and design.

Despite the unique characteristics of each of these ML-based algorithmic models, they also have limitations. First, these models often do not consider the heterogeneous information defined in relational networks. Second, AI/ML-based models require extensive training, and each application necessitates specific training tailored to its requirements. Additionally, shallow network- and sequential data-based approaches are usually insufficient to learn some of the key features (e.g., distance correlation) required to make accurate predictions (Wu et al., 2024).

### 2.3 DL-based algorithm

DL algorithms for drug discovery usually consist of convolutional neural networks (CNNs), generative adversarial networks (GANs), and recurrent neural networks (RNNs) (Figure 1). All of them play critical role in drug discovery and development, which have been summarized as following.

#### 2.3.1 CNNs

CNNs operate with a convolutional layer that slides over the original image using convolutional filters (typically small matrices of 3 × 3 or 5 × 5 in size), allowing each filter to extract specific features, thereby reducing the amount of computation and the risk of overfitting after maximum pooling and average pooling by a pooling layer. These are compressed into a lengthwise vector that serves as an input to a fully connected layer. The fully connected layer then uses these features to determine image categories. Lei et al. (2021) developed a learning framework based on DL called CAMP, which utilizes CNNs and self-attention mechanisms to adequately extract local and global information to predict binary interactions of input peptide-protein pairs.

#### 2.3.2 GANs

The operation of a GAN involves a generator and a discriminator. Random inputs are passed through the generator to produce new samples, which are then given to the discriminator to distinguish between real and fake. These two components continuously challenge each other to generate more authentic sample data (Gulakala et al., 2022). For instance, Wang et al. (2023) constructed a new CNN using dense networks. Dense networks perform multilayer transmission on the generator network of the GAN architecture, extending the training space and improving sequence generation efficiency (Wang et al., 2023).

#### 2.3.3 RNNs

RNNs are particularly important for analyzing information based on sequences or time series (Pandiyan and Wang, 2022), which are distinguished by their ability to process image and numerical data and learn data types exhibiting forward and backward correlations due to the network’s inherent ability to memorize them. Based on the advantages of RNNs for data processing, Sangrak et al. constructed an RNN model that significantly improved the performance of drug interaction extraction by incorporating positional features, subtree inclusion features, and integration methods. Compared to the top models in the same period, this model exhibited improved performance on the DDIExtraction Challenge’13 test data by up to 4.4% and 2.8%. This improvement provides an effective solution for large data processing and plays a key role in drug discovery (Lim et al., 2018).

Despite its widespread use, AI-based DL requires special conditions. It starts with the need for high-quality and sufficiently large data, which are often privately owned and not intuitively generated. Additionally, data in ML-generated “black boxes” can be difficult to interpret, especially in the fields of biology and chemistry (Lei et al., 2021).

## 3 AI opens a new chapter in drug screening

**FIGURE 2:**
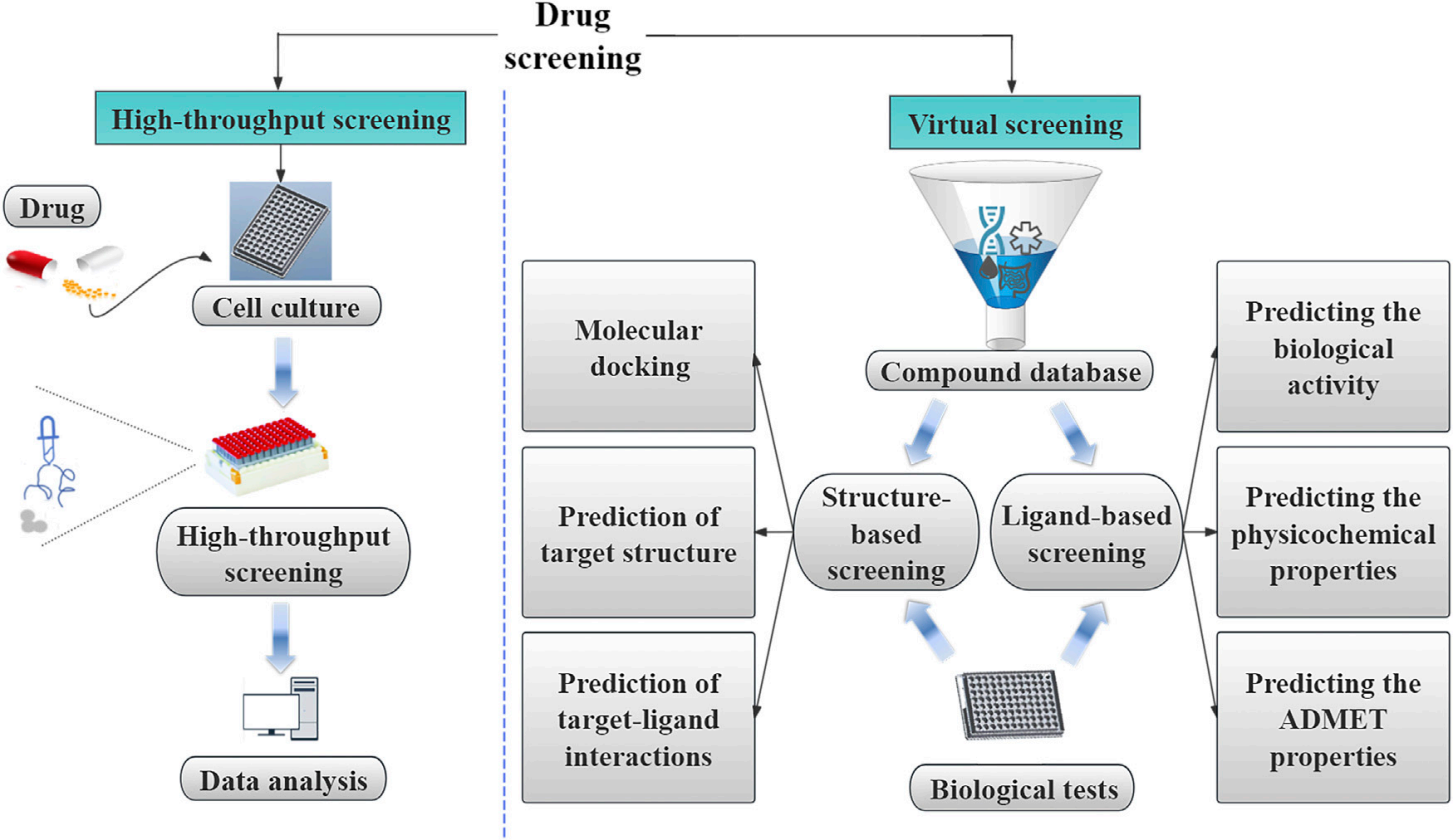
The categorization of drug screening into high-throughput screening and virtual screening. High-throughput screening is a classic method based on cell culture, high-throughput screening, and data analysis. Virtual screening is further divided into ligand-based and structure-based virtual screening. The text outlines the use of ligand-based virtual screening in the drug screening process.

Drug screening involves the identification and evaluation of the initial pharmacological properties of substances with potential medicinal applications aming to uncover their therapeutic value and clinical utility. This process is a foundational step in the research and development of novel drugs, providing essential data and insights. Drug screening is commonly divided into two main categories: high-throughput and virtual screening (Figure 2).

High-throughput screening uses experimental techniques at the cellular or molecular level, as well as microtiter plates, automated systems, and rapid detection instruments for data collection, analysis, and processing (Figure 2). This approach involves screening thousands or even millions of compounds during the drug discovery phase, serving as a crucial technique for identifying active compounds early in the drug development process (Figure 2) (Dueñas et al., 2023). For instance, in the context of ATR kinase inhibitor research, high-throughput screening using compound libraries has proven to be an effective strategy for discovering ATR kinase inhibitors, exemplified by the identification of the lead compound BAY-937 (Yuan et al., 2022). In antiviral research targeting articulator-associated protein kinase 1 (AAK1), Qi et al. used high-throughput RNAi screening to determine the pivotal role of AAK1 in regulating the entry of rabies virus into cells (Qi et al., 2022). Furthermore, in a study investigating the relationship between intestinal flora and Shengmai Yin, You et al. used quantitative sequencing technology to assess alterations in the composition of the intestinal microbial community in a rat model of splenic deficiency (You et al., 2020).

Virtual screening plays a crucial role in identifying potential drug candidates by evaluating and screening extensive structural libraries (Figure 2) (Singh et al., 2024). Using software tools, virtual screening enables the simulation of molecular interactions, calculation of affinities, and streamlining screening processes to improve efficiency. Virtual screening is particularly advantageous for drug discovery involving large numbers of small molecules, with libraries exceeding 10^60^ compounds within the total chemical space, surpassing the capacity of traditional high-throughput screening methods that are limited to tens to hundreds of thousands of compounds (Gorgulla et al., 2022). The two primary categories of virtual screening are ligand-based and structure-based virtual screening (Figure 2) (Singh et al., 2024; Parvatikar et al., 2023; Lin et al., 2022). By employing a hierarchical approach that combines ligand-based and structure-based virtual screening, researchers can sequentially apply filters to reduce the size of screening libraries to a manageable scale for experimental validation, yielding successful outcomes in numerous drug screening initiatives (Kumar and Zhang, 2015). Wei et al. used virtual screening in their investigation of transcriptome analysis to elucidate the regulatory mechanisms underlying the biosynthesis of warm tulipane terpenoids induced by methyl jasmonate. This approach, which involved the prediction of protein analyses and Hidden Markov Model mapping of terpenes, provided a faster and more efficient way of studying regulatory mechanisms than conventional methodologies (Wei et al., 2022).

Recently, particularly following the emergence of the novel coronavirus in 2019, vaccine and drug development timelines have been prolonged. In this unique context, the integration of AI in drug screening has garnered significant attention from researchers and scholars in the field of pharmacology. To address the challenges associated with drug screening, selecting appropriate algorithmic models to enhance the screening capabilities of AI has become a focal point of interest within the research community.

### 3.1 AI in ligand-based virtual screening

The ligand-based approach involves analyzing established active molecules and the prediction of their pharmacological properties by evaluating the resemblance of the compound under examination to the recognized active compounds (Liu R. et al., 2021). Estimating the binding affinity between a ligand and its target, known as drug-target binding affinity, is a crucial stage in ligand-based virtual screening. The objective of ligand-based virtual screening is to identify molecules with unique fundamental structures with similar or superior biologically active ligands compared to known biochemical activity when a known active ligand binds to the drug target, a phenomenon referred to as “scaffold hopping” (Böhm et al., 2004). Ligand-based AI techniques can predict not only the biological activity of compounds but also their physicochemical and pharmacokinetic characteristics.

#### 3.1.1 Predicting the biological activity of compounds

Determination of drug-target binding affinity is crucial for developing small-molecule drugs, as it indicates the strength of the interaction between a drug and its target (Sadybekov et al., 2022). Lack of affinity or interaction with non-target proteins can lead to ineffective therapeutic outcomes or potential toxicity. AI can help predict drug-target interactions by comparing drug and target characteristics to predict interactions, especially between similar drugs and targets (Öztürk et al., 2018). Currently, advanced models, including sequence-based, graph-based, and multimodal data-based models, are used; however, they face challenges in mining edge information, acquiring pharmacophore knowledge, integrating multimodal data, and simulating interactions (Zhang et al., 2023). To overcome these limitations, Li Zhang et al. introduced a method called graphical features and pharmacophore-enhanced cross-attention networks for drug-target binding affinity prediction. This method employs graph neural network (GNN) modules, linear projection units, and self-attention layers to extract features of drugs and proteins. Additionally, intramolecular and intermolecular cross-attention mechanisms were designed to merge and interact with the drug and protein features. Linear projection units were used to obtain the final drug and protein features, and the binding affinity of the drug to the target was predicted using a multilayer perceptron (Zhang et al., 2023).

#### 3.1.2 Predicting the physicochemical properties of compounds

Physicochemical characteristics indirectly influence the pharmacokinetic attributes and target receptor families of pharmaceuticals, making them crucial in the discovery and development of novel drugs (Zang et al., 2017). Currently, most techniques used to evaluate the physicochemical properties of drugs are based on traditional trial-and-error approaches. However, more sophisticated methods have been introduced as a result of recent advancements, such as six predictive models (SVMs, ANNs, lncRNA-disease associations, probabilistic neural network algorithms, kNN algorithms, and partial least squares) developed by researchers like Kumar (Kumar and Kim, 2021). These models were based on cumulative parameters like molecular refractive index, molecular volume, molecular surface area, molecular weight, log P, log S, and total polar surface area and have been used to predict the intestinal absorption of 497 compounds.

#### 3.1.3 Predicting the absorption, distribution, metabolism, excretion, and toxicity tolerance (ADMET) properties of compounds

A successful drug should not only exhibit strong biological activity and favorable physicochemical characteristics but also possess excellent ADMET properties and undergo efficient pharmacokinetic processes. The inadequate pharmacokinetic attributes and potential toxicity of candidate compounds are significant contributors to the failure of drug development endeavors. The efficacy of targeted cancer therapies, a burgeoning antitumor treatment modality, necessitates drug delivery systems with minimal immunogenicity and toxicity levels (Luo et al., 2021). AI can be used to predict drug toxicity by analyzing the chemical structures and properties of compounds. ML algorithms, trained on toxicology databases, can predict detrimental effects and identify hazardous structural attributes. This predictive capacity aids researchers in prioritizing safer chemicals and mitigating adverse effects during clinical trials. For instance, Xiong et al. (2021) introduced the ADMETlab model, founded on *in silico* ADMET and is based on version 2.0 constructed using the Python Web framework Django. This model, hosted on the AliCloud Ubuntu Linux system, offers an expanded range of ADMET endpoints than its predecessor, including 17 physical chemistry, 13 medicinal chemistry, 23 ADME characterization, 27 toxicity endpoints, and 8 toxicogenic rules.

### 3.2 Utilization of AI in structure-based virtual screening

The application of AI in structure-based virtual screening, also known as target-based virtual screening, has become prevalent in the prediction of target-ligand interactions (Parvatikar et al., 2023). Structure-based virtual screening involves using molecular docking based on the three-dimensional (3D) structure of proteins to analyze the characteristics of target protein-binding sites and their interactions with small-molecule drugs. The binding affinity of the proteins to the drugs was evaluated using affinity scoring functions. Drugs exhibiting high predictive scores were selected from a vast pool of compound molecules for subsequent bioactivity testing. Molecular docking is the primary method used in this process (Singh et al., 2024). It is common to combine molecular docking with AI algorithms to validate docking outcomes and further refine compound screening (Lin et al., 2022). In contrast to ligand-based virtual screening, structure-based virtual screening can identify ligands with novel scaffolds or chemical functional groups (Dilip et al., 2016). For instance, fragments derived from natural products can be valuable lead compounds for the design and discovery of small-molecule drugs (Bai et al., 2021). The modification or simplification of natural product scaffolds is a key strategy in natural product drug discovery, and structure-based virtual screening can provide novel insights into this domain (Hui et al., 2024).

#### 3.2.1 Molecular docking

Molecular docking is a computational technique used to predict the preferred orientation and structure of a ligand molecule when it interacts with another molecule, typically a larger receptor (Cerqueira et al., 2015). For instance, DeepDock, an AI-driven molecular docking approach, leverages deep learning algorithms to forecast binding modes and affinities in molecular docking scenarios. Unimol utilizes graph neural network technology to predict docking outcomes with enhanced efficiency. In addition, DiffDock amalgamates deep learning with graphical convolutional networks, thereby augmenting the accuracy and velocity of docking predictions (McNutt et al., 2021; He et al., 2024; B Fortela et al., 2024). This method is commonly used to predict target-ligand interactions (Pinzi and Rastelli, 2019). For instance, Yan et al. conducted molecular docking using Discovery Studio to evaluate the binding affinity of Mauritania with its potential targets, identifying PIK3CA as a promising target (Yan et al., 2022). In another study, Discovery Studio was used for molecular docking to identify the potential binding site of SIRT3 with craniospermone, revealing novel roles and mechanisms of craniospermone in anti-glomerular basement membrane antibody disease (GBM) and suggesting a new therapeutic approach for GBM treatment (Sun et al., 2022). Additionally, Gao Y et al. conducted molecular docking studies using the crystal structure of hHDAC1 to investigate the binding modes of compounds 27f and 39f to HDAC1 and HDAC6, demonstrating that the synthesized HDAC inhibitors 27f and 39f exhibited favorable binding modes and elucidated their potent inhibitory effects against HDAC1 and HDAC6 (Gao et al., 2023).

#### 3.2.2 Predicting target structure

The prediction of the target structure plays a pivotal role in drug screening. Using AI, target protein structures can be predicted and evaluated to provide crucial insights for drug screening, thereby improving the efficiency of research and development endeavors. In a study of N^6^-methyladenosine (m^6^A), Liu et al. (Sui et al., 2020; Liu S. et al., 2023)established a novel database named “M6AREG” to facilitate the screening of drug-target interactions. They used crosslinking techniques and other methodologies to improve data collection, aiming to advance the future development of m^6^A research. Additionally, Can et al. identified potential peptide biomarkers within the Dendrobium genus using an AI-driven multivariate statistical analysis approach (Fang et al., 2020). Google’s DeepMind introduced AlphaFold, a tool that leverages AI technology to train on Protein Data Bank structural data to predict the 3D structure of amino acid sequences (Powles and Hodson, 2017). The updated version of AlphaFold, AlphaFold2, integrates a novel graph neural network to improve the accuracy of target protein structure prediction (Lv et al., 2023). Although this method offers advantages in predicting target structures, there are areas of uncertainty (Meller et al., 2023). Therefore, new models, like the AlphaFold-multitimer model, have been developed and used to significantly improve the accuracy of predicting multichain protein structures (Lv et al., 2023). Furthermore, the development of the MULTICOM four-level structure prediction system improved the AlphaFold-multitimer model’s ability to predict structurally intricate proteins (Liu J. et al., 2023). The latest iteration AlphaFold 3, which has a notable enhancement in DL algorithms and model architecture over its predecessors, allows for more effective management of intricate protein structures and extensive datasets. It is anticipated to achieve greater precision in the 3D structure prediction of proteins, particularly those that are complex and varied. The refined algorithms and computational infrastructure of AlphaFold 3 are designed to substantially decrease the prediction time. Additionally, the platform now encompasses advanced functionalities such as the prediction of protein-protein interactions and the binding of proteins to ligands (Jumper et al., 2021). However, protein folding remains a challenge for this approach (David et al., 2022). DN-fold, a DL technique for predicting protein-folded structures, has been proposed to address the prediction challenges encountered by AlphaFold (Jo et al., 2015). These examples underscore the notable achievements of AI-based target structure prediction, which has the potential to significantly improve the efficacy of drug discovery and development processes.

#### 3.2.3 Predicting target-ligand interactions

The interactions between targets and ligands are intricate, and investigating their operational patterns can establish a theoretical foundation for drug screening. Using AI technology to analyze, assimilate, and prognosticate extensive datasets concerning known drug-receptor interactions can facilitate a comprehensive understanding of the mechanism of action and influential variables, thereby ensuring a logical and efficient drug screening process. Scoring functions are essential for predicting the binding affinities of drugs and targets (Selvaraj et al., 2022). The DL-based scoring function hinges on extracting features (such as distance and charge) from a representation of receptor-ligand interactions and subsequently predicting the binding affinity between the two entities (Kumar and Kim, 2021). Several scholars have combined RF and AutoDock scoring functions with the Glide XP score to achieve superior scoring outcomes (Brown et al., 2021). Jiménez et al. (2018) developed a 3D graph-based CNN model to predict the interaction between ligand and receptor proteins, with their projected binding affinities closely aligning with the experimental data. Wang et al. developed a multiscale convolutional model capable of capturing the receptor and ligand characteristics to predict their interactions (Wang et al., 2021). Furthermore, Jin et al. (2021) introduced an EmbedDTI model, which improved the depiction of ligands and receptors while bolstering drug-target interaction prediction.

In the field of protein-protein interactions, numerous potential drug targets exist, and the process of discovering and identifying these targets is crucial for drug screening. For instance, in cancer therapeutic research, the use of SwissTargetPrediction for Muritan target prediction led to the identification of calmodulin as a promising target for lung cancer treatment (Chen et al., 2020). Furthermore, advancements in the high-resolution crystal structures of KDM1A inhibitor complexes have provided researchers with valuable insights into protein-ligand interactions, facilitating the screening of inhibitors with enhanced selectivity (He et al., 2022). Additionally, by investigating natural products, including flavonoids, alkaloids, and terpenoids, and their interactions with target proteins, scientists have gained insights into their ability to selectively target and inhibit inflammatory mediators, leading to the development of effective anti-inflammatory strategies (Li et al., 2023).

## 4 AI-powered drug design

The use of ML and DL algorithms in the field of drug development can facilitate the understanding of the intricate interplay between chemical and biological data (Mervin et al., 2021). Recently, computer-aided drug design (CADD) has gained significant traction in the pursuit of novel pharmaceuticals (Singh et al., 2024). Leveraging the computational capabilities of these algorithms has broadened their accessibility and reduced their costs. For example, Jiang XY et al. used CADD to investigate the structural modifications, monosubstituted derivatives, disubstituted derivatives, and disubstituted polymers of β-elemene, leading to improved hydrophilicity and antitumor activity. In addition to predicting the target structures and receptor-ligand interactions, CADD facilitates the *de novo* design of novel active compounds and the automated synthesis of drugs, thereby addressing challenges in organic synthesis (Jiang et al., 2024).

### 4.1 Redesigning active molecules

Creating novel active molecules from scratch represents a significant advancement in drug discovery and development. Computer-aided *ab initio* design to develop new active molecules has emerged as a valuable tool for drug design innovation (Reker et al., 2014). This approach involves using computational simulations and ML techniques to design bioactive molecular structures from first principles, aiming to address the scarcity of new chemical entities in drug discovery and cater to the demands of effective drug therapy (Reker et al., 2014).

Currently, there have been significant advancements in molecular design methodologies, including the use of autoencoders, GANs, and RNNs. Among these techniques, the variant autoencoder method stands out, comprising an encoder network and a decoder network (Gómez-Bombarelli et al., 2018). This method facilitates the transformation of chemical structures represented by the simplified molecular input line entry system (SMILES) notation into continuous real-valued vectors through the decoder. These continuous vectors serve as a potential space, with the decoder converting them back into chemical structures (Hessler and Baringhaus, 2018). Researchers have successfully used this method to train a model based on the quality estimation of drug-likeness ratings and synthetic accessibility score, generating more targeted molecules (Hessler and Baringhaus, 2018).

Furthermore, Kadurin et al. (2017) used GANs to propose novel molecules with potential anticancer properties. Additionally, RNNs have demonstrated promise in the *ab initio* design of drugs. RNNs are trained to encode chemical structures by generating numerous SMILES strings through training on a variety of compounds from different compound libraries. This approach has demonstrated the ability to generate novel peptide structures, demonstrating the potential of RNNs in drug design innovation (Hessler and Baringhaus, 2018).

### 4.2 Addressing the difficulties in organic synthesis and achieving the automation of drug synthesis

Addressing of challenges encountered in organic synthesis is a significant obstacle in the field of drug development. AI technology is crucial in predicting the reaction processes and optimizing synthetic pathways to facilitate rapid organic synthesis and drug design. The ML-based methods for forward synthesis prediction can accurately determine the sequence of synthetic routes and predict reactions and products (Hessler and Baringhaus, 2018). For example, PathPred (Table 1) is a robust web server capable of predicting multi-step synthetic pathways for a given compound, thereby enhancing the efficiency and speed of organic synthesis (Molnar et al., 2022). Liu et al. developed a model for retrosynthesis prediction that uses ML techniques and extensive datasets to accurately predict chemical reactions, thereby addressing the challenges of organic synthesis (Liu et al., 2017). Furthermore, AI enables the automation of compound synthesis (Mouchlis et al., 2021), as demonstrated by the development of Chemputer (Tripathi M. K. et al., 2021) a computer-aided software designed for automated compound synthesis.

**TABLE 1:** AI based drug discovery and development tools and software.

Number	Drug discovery software based on AI	Auxiliary direction	Advantage
1	DeepDrug (Yin et al., 2023)	Detecting the genome of pathogens	Can access very large datasets and quickly identify new compounds
2	Reinvent (Blaschke et al., 2020)	Mainly used for molecular generation	Can conduct AI molecular design
3	AlphaFold (Nussinov et al., 2022)	Predicting the structure of proteins	Help understand the interaction between drugs and targets
4	SwissADME (Daina et al., 2017)	Predicting the oral bioavailability of compounds, etc	Quickly screen potential drug candidates in the early stages of drug development
5	ADMETSAR (Yang et al., 2018)	Predicting the toxicity of drug molecules	Plays an important role in drug development and safety evaluation
6	AutoDock Vina (Santos-Martins et al., 2019)	Determine binding conformation and binding affinity	Having high accuracy and relatively high docking speed
7	ADMETsar (Cheng et al., 2019)	Predicting the properties of compounds	Efficient, fast, and can reduce repeated experiments
8	DeepPurpose (Huang et al., 2021)	Drug activity prediction	Easy to operate and flexible to use
19	MATLAB (Molnar et al., 2022)	Assist in exploring the potential characteristics of drugs	The algorithm is efficient and highly targeted
10	PathPred (Ma et al., 2020)	Predicting the synthetic route of compounds	Can conveniently and intuitively demonstrate the synthetic route of compounds
11	Deep 6 AI (Lei et al., 2024; Liu et al., 2022)	Assist in quickly identifying potential trial participants	Improve patient recruitment efficiency

## 5 AI’s revolutionary role in drug clinical trials

Clinical trials are crucial for evaluating the safety, efficacy, and reliability of drug development processes. These trials follow a standardized, sequential methodology where scientists assess the safety, efficacy, and clinical relevance of promising new drugs (Bhavya et al., 2023; Vasan et al., 2023). However, clinical trials are labor-intensive and involve patient recruitment, enrollment, continuous monitoring, medical adherence, and data retention. Personalized AI solutions can streamline and expedite these experiments by managing trial data, integrating patient histories, and focusing on patient-centered AI approaches (Chopra et al., 2023). The advent of AI has revolutionized the data collection and monitoring aspects of clinical trials, leading to reduced costs, increased efficiency, and improved drug development research. The ways in which AI can contribute to pharmacy clinical trials, including its applications in participant recruitment, data collection and analysis, predictive analytics for trial design, and patient monitoring and safety, were summarized in this study.

### 5.1 Improving participant recruitment efficiency

In phase I clinical trials, approximately 80% of trials experience delays in patient enrollment (Chopra et al., 2023). The recruitment of suitable trial participants is a time-consuming and costly aspect of clinical trials. Traditional methods of participant recruitment in clinical trials involve professionals manually screening extensive medical records, posing challenges in terms of both quantity and quality (Lovato et al., 1997; Britton et al., 1999). Conversely, AI technology can quickly identify potential participants meeting specific trial criteria by analyzing electronic health records, social media platforms, and other online data sources. For instance, Deep 6 AI (Table 1) has developed a technology capable of sifting through millions of patient records to quickly identify suitable clinical trial candidates. Similarly, Mendel. AI uses an AI system for precise matching of medical records with clinical trial data, ensuring prompt notification to patients to promote diversity and representativeness in study samples (Lei et al., 2024; Liu et al., 2022).

### 5.2 Improving data collection and analysis

Conventional methods for collecting and evaluating data from drug clinical trials exhibit various limitations, including reduced data efficiency, constrained utilization, susceptibility to errors, limited scalability, and inadequate monitoring (Jüni et al., 1999). Conversely, AI provides a solution to these challenges. AI plays a crucial role in improving the quality and expediency of data collection and analysis in drug clinical trials by effectively processing and conducting in-depth analyses of extensive and intricate datasets. ML techniques excel in managing and analyzing large datasets from clinical trials, enabling the identification of overlooked issues and concealed risks. Furthermore, AI can monitor real-time data from wearable devices to monitor participants’ health metrics, thereby providing researchers with accurate and timely information (Lei et al., 2024; Tripathi N. et al., 2021; Kolluri et al., 2022)

### 5.3 Predictive analytics applied to trial design

AI-powered predictive analytics are instrumental in the trial design phase as they aid in predicting the potential outcomes of a clinical trial based on the analysis of historical trial data and other relevant information (Fleming and DeMets, 1996). By leveraging AI, researchers can predict the efficacy of drug treatments before conducting trials, enabling informed decisions regarding trial design, such as determining the optimal drug dosage and the early detection of potential side effects (Aliper et al., 2023).

### 5.4 Improved patient monitoring and safety measures

Furthermore, AI-based monitoring tools are crucial in improving patient safety by automatically collecting data to promptly identify and address safety concerns. Moreover, AI technology can assist in monitoring patient adherence to trial protocols, thereby ensuring the credibility and accuracy of trial outcomes (Moazemi et al., 2023).

## 6 Challenges of AI in drug development

AI has demonstrated improved outcomes across various stages of drug development. However, significant challenges remain in this domain. First, the intricate nature of drug actions within an organism presents a complex hurdle (Yu et al., 2023). The failure of certain drugs to progress to clinical trials can be attributed to the fact that AI-driven drug development processes are conducted in a controlled environment that lacks the complexity of real-world conditions (Yu et al., 2023). Second, the efficacy of AI models in drug screening is influenced by the quality and diversity of research data. Moreover, AI relies significantly on data and specialized personnel. Additionally, concerns arise when technologies like facial recognition software or other ML tools are used to monitor trial participants, potentially infringing upon their privacy (Almeida et al., 2022). Furthermore, AI is susceptible to issues like programming errors that can lead to missed opportunities in drug studies. These challenges are not easily surmountable in the short term. The integration of AI in drug research not only tests the limits of time and technology but also represents humanity’s quest into uncharted territories, signifying a long and arduous journey ahead.

## 7 Prospect

Integration of AI is becoming more common in the field of drug development. The current era is characterized by a surge in AI technologies, exemplified by the introduction of the text-to-image model Dall-E in 2021 and its successor Dall-E2 in 2022 (Adams et al., 2023). These tools are considered promising for image generation, enhancement, and manipulation in future radiology AI research. The release of ChatGPT was succeeded by GPT-4, demonstrating its significant potential for application in pharmacology, spanning from research topic identification to clinical laboratory diagnosis (Dave et al., 2023). Recently, OpenAI formally introduced the text-to-video model Sora, which is currently in the feedback acquisition phase. Despite being in the early stages of development, Sora’s performance and potential are comparable to that of GPT-4. It is anticipated that Sora will soon be used in drug discovery. The integration of AI technology into drug development and mining endeavors is poised to make a significant and groundbreaking impact on human health and wellbeing.
